# Comparing biweekly single-dose actinomycin D with multiday methotrexate therapy for low-risk gestational trophoblastic neoplasia (FIGO Score 0–4): study protocol for a prospective, multicentre, randomized trial

**DOI:** 10.1186/s12885-023-11225-2

**Published:** 2023-08-23

**Authors:** Fang Jiang, Ming-yi Mao, Yang Xiang, Xin Lu, Chong-li Guan, Lan-zhou Jiao, Xi-Run Wan, Feng-Zhi Feng, Tong Ren, Jun-Jun Yang, Jun Zhao

**Affiliations:** 1 Department of Obstetrics & Gynaecology, Peking Union Medical College Hospital, Peking Union Medical College, Chinese Academy of Medical Science, National Clinical Research Center for Obstetric & Gynaecologic Diseases, No. 1 Shuaifuyuan Wangfujing Dongcheng District, Beijing, 100730 China; 2 Department of Gynecology Oncology, Obstetrics and Gynecology Hospital of Fudan University, Shanghai, China; 3 Department of Gynecology Oncology, Gansu Provincial Maternity and Child-Care Hospital, Lanzhou, Gansu Province China; 4 Department of Gynecology Oncology, Dalian Women’s and Children’s Medical Center (Group), No.1 Dunhuang Road Shahekou, Dalian, Liaoning China

**Keywords:** Gestational trophoblastic neoplasia, Actinomycin D, Methotrexate, Low risk

## Abstract

**Background:**

Single-agent chemotherapy using methotrexate or actinomycin D is the first-line treatment for patients with low-risk gestational trophoblastic neoplasia. Various methotrexate-based and actinomycin D-based single-agent regimens can be used. However, there is insufficient evidence to determine the superior regimen. To guide doctors in selecting a single-agent chemotherapy regimen for patients with low-risk gestational trophoblastic neoplasia, we will compare two regimens.

**Methods:**

We will conduct a multicentre, randomized, prospective clinical trial. Selected low-risk gestational trophoblastic neoplasia patients (FIGO score 0–4) will be randomized 1:1 to a biweekly single-dose actinomycin D group or a multiday methotrexate therapy group. The actinomycin D group will receive IV pulse actinomycin D (1.25 mg/m^2^) every 14 days, and the methotrexate group will receive methotrexate (50 mg) intramuscularly on days 1, 3, 5, and 7 (4 doses per cycle) and leucovorin (15 mg) intramuscularly on days 2, 4, 6, and 8. This process will be repeated every 14 days. The primary endpoints will include the complete remission rate by single-agent therapy and the overall complete remission rate. The secondary endpoints will include the duration needed to achieve complete remission after single-agent chemotherapy, number of courses needed to achieve complete remission after single-agent chemotherapy, incidence and severity of adverse effects, effects on menstrual conditions and ovarian function based on the anti-Mullerian hormone level, and patient-reported quality of life.

**Discussion:**

Previous clinical trials comparing biweekly single-dose actinomycin D with multiday methotrexate therapy for treating low-risk gestational trophoblastic neoplasia patients failed to meet the expected case number. Through this multicentre study, the complete remission ratio and efficacy difference between biweekly single-dose actinomycin D and multiday methotrexate therapy will be obtained. This study will also provide the basis for formulating a preferred regimen for treating patients with low-risk gestational trophoblastic neoplasia.

**Trial registration:**

ClinicalTrials.gov: NCT04562558, Registered on 13 September 2020 (Protocol version 2020–9-24, version 1.0).

## Background

Gestational trophoblastic neoplasia (GTN) comprises a group of pregnancy-related tumours, including invasive mole, choriocarcinoma, placental site trophoblastic tumour (PSTT), and epithelioid trophoblastic tumour (ETT).

GTN is staged using the International Federation of Gynecology and Obstetrics (FIGO) 2000 staging and prognostic scoring system, which was promulgated in 2002 and is currently still used. In this scoring system, a risk score of 6 and below is classified as low risk. According to the FIGO prognostic scores, treatment of GTN depends on stage and is personalized [1]. Patients with low-risk GTN (risk score 0–4) should be treated with single-agent chemotherapy. Commonly used drugs include methotrexate (MTX) and actinomycin D (Act-D).

There are many different single-agent chemotherapy regimens for patients with low-risk GTN. These regimens are different in drug dose, frequency and mode of administration. Four MTX regimens are commonly used: (1) an MTX 8-day regimen (50 mg MTX administered intramuscularly on days 1, 3, 5, and 7 and 15 mg leucovorin administered orally/ intramuscularly 24 h after MTX on days 2, 4, 6, and 8) repeated every 2 weeks; (2) an MTX dose of 0.4 mg/kg (max. 25 mg) administered intravenously or intramuscularly for 5 days every 2 weeks [2]; (3) an MTX dose of 30–50 mg/m^2^ administered intramuscularly weekly; and (4) an MTX 300 mg/m^2^ infusion administered over 12 h every 2 weeks. Common regimens for Act-D include a 5-day regimen (0.5 mg Act-D administered intravenously for 5 days every 2 weeks) and a pulse regimen (1.25 mg/m^2^ Act-D pulse administered intravenously every 2 weeks) [3]. There is no high-level evidence to support which regimen is the best. Lawrie et al. [4] conducted a meta-analysis of 7 randomized clinical trials (RCTs) in 2016. The authors suggested that Act-D treatment was probably more likely to achieve an initial cure and less likely to result in treatment failure than MTX (RR 0.65, 95% CI 0.57 ~ 0.75). They found little or no difference in the side effects between Act-D and MTX. In 2018, Li et al. [5] performed a network meta-analysis based on the same 7 RCTs and another 4 retrospective studies including 987 patients. This network meta-analysis showed that Act-D-based regimens were superior to MTX-based regimens and that 5d-IV Act-D might be the best regimen, followed by pulse IV Act-D and 5d-IV MTX. In the 2020 National Comprehensive Cancer Network (NCCN) guidelines, a multiday MTX regimen is typically used as first-line therapy for patients with low-risk GTN. At present, the complete remission (CR) rates of patients with treatment-naive low-risk GTN vary greatly when different treatment regimens are applied. For single-agent MTX-based regimens, the CR rates are 69–80%. The results of the meta-analysis showed that weekly MTX had a poor effective rate. The MTX 8-day regimen was introduced in 1979 [6]. After exploring the dose of chemotherapy used, it was found that increasing a single dose of MTX had no effect on treatment [7]. Comparing patients receiving 50 mg total dose/day or 1 mg/kg/day intramuscular MTX, there was no difference in the average number of chemotherapy courses required to achieve CR (5.7 ± 2.6 vs. 6.3 ± 2.3, *p* = 0.106) or in the rates of MTX resistance (28.3% vs. 22.1%, *p* = 0.387) and relapse (3% vs. 6.5%, *p* = 0.300). Subgroup analysis stratifying patients by weight confirmed these results. Thus, a fixed dose of 50 mg MTX administered intramuscularly on days 1, 3, 5, and 7 is now widely used in Europe, with a CR rate of 69–89% for patients with low-risk GTN. For single-agent Act-D-based regimens, CR rates for pulse regimens vary between 69% and 90%. There are limited studies comparing the efficacy of the multiday MTX regimen with the biweekly single-dose Act-D regimen. Only one prospective study (NCT01535053) [8] showed that the complete response rates for the two regimens were 88% and 79%, respectively, and that the side effects of MTX were greater (*p* = NS). However, the sample size of this study did not reach the design size. Thus, this study failed to show which regimen was better and was unable to demonstrate that the MTX regimen was not inferior to the Act-D regimen.

Therefore, the selection of single-agent chemotherapy regimens for low-risk GTN patients requires high-level evidence. Currently, in most hospitals in China, Act-D single-agent chemotherapy is more commonly used, and based on international reports, the multiday MTX regimen has not shown effectiveness. Thus, the purpose of this study will be to analyse single-agent therapy strategies for low-risk GTN patients (0–4 points). We will compare the efficacy and safety of the pulse Act-D regimen that is well recognized in the literature and the MTX 8-day regimen widely used in Europe and North America. This prospective randomized controlled study will be conducted at multiple centres in China, which will provide more convincing results and significance for the selection of a first-line single-agent chemotherapy regimen for patients with low-risk GTN.

## Methods

### Study design

This study is designed as a prospective, multicentre, randomized clinical trial with two parallel arms to test the hypothesis that treatment with multiday MTX is inferior to treatment with biweekly single-dose Act-D in patients with low-risk gestational trophoblastic disease based on complete response results. We will also compare the toxicity of these regimens in the two treatment groups.

**Fig. 1:**
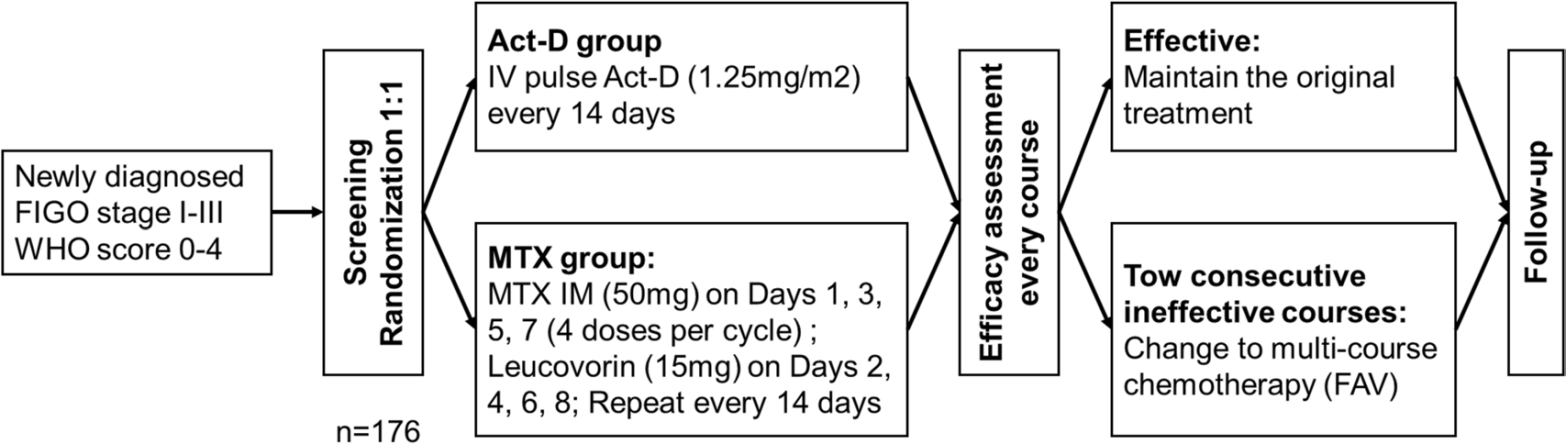
Graphical representation of the study design. Act-D, actinomycin D; IV, intravenously; MTX, methotrexate; IM: intramuscularly; FAV, fluorouracil/floxuridine, actinomycin-D, vincristine

The study design is summarized in Fig. 1 and Table 1. All patients will be randomized in a 1:1 ratio to the biweekly single-dose Act-D group or the multiday MTX therapy group. The primary outcomes are the single-agent CR rate and the overall CR rate, which is defined as the percentage of participants with complete response after single-agent chemotherapy and those after second-line multiple-drug chemotherapy after single-agent failure. The secondary outcomes are the time to achieve CR after single-agent chemotherapy, the number of courses needed to achieve CR after single-agent chemotherapy, the incidence and severity of adverse effects (grade 3 or higher), the effects on menstrual conditions and ovarian function based on the anti-Mullerian hormone (AMH) level, and patient-reported quality of life (QOL). Adverse effects will be assessed by Common Terminology Criteria for Adverse Events (CTCAE) Version 5.0.

**Table 1:** The schedule of enrolment, interventions, and assessments

**TIMEPOINT**	**STUDY PERIOD**					
	**Enrolment**	**Allocation**	**Treatment period**			**Follow-up period**
	***Day -14***	***Day* 0**	***Per cycle(single-agent)***	***Second-line chemotherapy (first course)***	***Second line and consolidation chemotherapy***	***Every 12 weeks***
**ENROLMENT**						
**Eligibility screen**	X					
**Informed consent**	X					
**Demographic information**	X					
**Medical history**	X					
**Allocation**		X				
**INTERVENTIONS**						
**Actinomycin D/ Methotrexate**			X		X	
**Multi-course chemotherapy (EMA-CO)**				X	X	
**ASSESSMENTS**						
***Tumor assessment (CT/MRI)***	X			X	X (if foci)	X
***Blood hCG***	X		X	X	X	X (every 2 weeks)
***EORTC QLQ-C30***	X			X	X (after the last course)	X
***Concomitant medication and treatment***	X		X	X		X
***ECOG***	X					X
***Adverse reaction of chemotherapy***	X		X	X	X	
***Physical examination***	X		X	X	X	X
***AMH***	X			X	X (after the last course)	X
***Routine blood test***	X		X	X	X	X (every 2 weeks)
***Blood biochemical full set***	X		X	X	X	X

### Patient recruitment and screening

A total of 228 patients with low-risk GTN will be recruited from 8 centres in China, including Peking Union Medical College Hospital, Dalian Maternal and Child Health Care Hospital & Dalian Obstetrics and Gynecology Hospital affiliated with Dalian Medical University, Shanghai Red House Obstetrics & Gynecology Hospital, Liaoning Cancer Hospital & Institute, Beijing Obstetrics and Gynecology Hospital, the Chemotherapy Center of Gansu Provincial Maternity and Child-care Hospital, Taiyuan Maternity and Child-Care Hospital, and Fujian Maternal and Child Health Hospital. All eligible patients will be informed of the trial at each centre. Written informed consent will be obtained from each patient prior to randomization.

### Inclusion criteria

The inclusion criteria are as follows:

1. Clinically proven low-risk GTN (persistent hydatidiform mole or choriocarcinoma) defined as 1 of the following:
  - Less than 10% decrease in the beta human chorionic gonadotropin (hCG) titre over 3 weeks
  - Greater than 20% sustained rise in the beta hCG titre over two consecutive weeks
  - Histologically proven choriocarcinoma or invasive mole
2. Stage I—III disease
3. WHO score 0–4
4. Age 18–70 y, female
5. No prior chemotherapy for GTN
6. Signed informed consent
7. Performance status—EGOG 0–2
8. Laboratory examination: WBC ≥ 3.5 × 10^9^/L, granulocyte count ≥ 1.5 × 10^9^/L, platelet count ≥ 80 × 10^9^/L, serum bilirubin ≤ 1.5 times the upper limit of normal, transaminase ≤ 1.5 times the upper limit of normal, blood urea nitrogen, creatinine ≤ normal
9. Fertile patients must use effective contraception during and for one year after study entry.

### Exclusion criteria

The exclusion criteria are as follows:

1. Histologically confirmed placental-site trophoblastic tumour (PSTT) or epithelioid trophoblastic tumour (ETT)
2. Primary choriocarcinoma
3. WHO risk score > 4
4. Previous MTX treatment for suspected ectopic pregnancy
5. Severe or uncontrolled internal disease, unable to receive chemotherapy
6. Concurrent participation in other clinical trials
7. Unable or unwilling to sign informed consent
8. Unable or unwilling to abide by the protocol.

### Randomization

A random number list will be generated using SAS 9.0 to randomize participants either to the Act-D group or the MTX group in a 1:1 ratio. After all eligibility criteria are met and written informed consent is obtained, the patients will obtain their enrolment numbers according to the order of enrolment. Allocation is kept in sealed and consecutively numbered envelopes. Patients, physicians, researchers, will be blinded before randomization. Then, they will be assigned to either the Act-D group or the MTX group according to the random number list.

### Intervention

Patients in the Act-D group will receive IV pulse Act-D (1.25 mg/m^2^) every 14 days (2 mg max dose). Patients in the MTX group will receive MTX intramuscularly (50 mg) on days 1, 3, 5, and 7 (4 doses per cycle) with leucovorin (15 mg) on days 2, 4, 6, and 8. This process will be repeated every 14 days. Patients in both groups will continue on treatment until the beta hCG titre is below the institutional normal level. Patients then will receive 2–3 additional consolidation treatments. If the level of hCG becomes stationary for at least 2 courses of single-agent chemotherapy or rises again, the patient will be referred to multicourse chemotherapy. FAV (fluorouracil/floxuridine, actinomycin-D, vincristine) is the first choice. If FAV is not available, the alternative can be EMA-CO (etoposide, MTX, Act-D/cyclophosphamide, vincristine).

During chemotherapy, patients will be monitored for adverse effects, including routine blood test, blood biochemical full set, etc. If the routine blood test shows grade 3 and 4 myelosuppression, patients will be given GCSF. If patients can recover to lower than1 grade myelosuppression, chemotherapy will continue. In case of liver and kidney function show 3 and 4 adverse reactions, intravenous liver-protecting drugs will be used, and treatment will be continued after recovery.

After completing chemotherapy, participants will be informed the follow-up period and the date for the next appointment. If participants are unable to visit the hospital where they received chemotherapy due to the COVID-19, they can choose the nearest hospital for examination, and send the results to the doctor by email or WeChat. The results of some examinations that are unable to complete will be recorded as missing data. If a participant fails to respond, we will try to regain contact via telephone, email and WeChat. A minimum of 3 attempts will be made to contact patients before they are considered to be lost to follow-up. The reasons for withdrawal will be documented carefully. Any data on a subject’s participation and procedures prior to withdrawal will be analyzed within the research.

### Sample size calculation

According to the efficacy of the two chemotherapy regimens reported in previous literature, we estimated that the CR rates of biweekly single-dose Act-D and multiday MTX therapy are 78% and 60%, respectively. Conducting a randomized controlled study with an allocation ratio of 1:1 and considering α = 0.05 and power = 0.8, 103 patients per group are needed. Considering the 10% drop-out rate, each group requires 114 patients (228 patients in total).

### Statistical analyses

All analyses will be performed using SPSS 20.0. T tests will be used to assess normally distributed data, and the results will be expressed as the $\overline{x }$ ± s. When the data do not conform to a normal distribution, the Mann–Whitney test will be used, and the results will be expressed as the [M(P25 ~ P75)]. Enumeration data will be expressed by case number or percentages and analysed by using the χ2 test. Grade data will be compared using the rank sum test. A *p* value of < 0.05 will indicate statistical significance. Kaplan–Meier and Cox proportional hazards regression analyses will be used for survival analyses. Factors with a *p* value < 0.1 by univariate analysis will be included in a multivariate Cox proportional hazards regression analysis. The last observation carried forward will be used to handle missing values.

### Data management

A specialized statistician will establish a dedicated electronic database to host the data. The study team will provide a unified case report form (CRF), and researchers will fill out the CRF. The participants’ information added by each centre will be fed back to the organizer regularly. All the CRFs will be kept by the research principal investigator. All the electronic data will be double entered to minimize data entry errors. Access to the electronic database will be limited to the principal investigator and statistician to maintain confidentiality.

## Discussion

GTN is divided into high-risk and low-risk groups according to the FIGO 2000 clinical stage and prognostic scoring system. According to the FIGO guidelines, single-agent chemotherapy is recommended for low-risk patients. However, there are currently many single-agent options, and there is a lack of high-level evidence regarding whether the MTX or Act-D regimen is better. In the current study, we aimed to address the lack of high-level evidence regarding the optimal single-agent chemotherapy regimen for low-risk gestational trophoblastic neoplasia (GTN) patients. Therefore, the purpose of this study was to design a prospective, multicentre randomized controlled clinical trial to compare the efficacy and safety of the MTX 8-day regimen and biweekly single-dose Act-D regimen.

In our study, we acknowledge the different methotrexate (MTX) regimens used in clinical practice, such as the 1 mg/kg MTX regimen commonly used in North America [9] and the 50 mg fixed-dose MTX regimen prevalent in Europe. While recognizing the potential impact of patient weight, particularly in overweight or obese individuals, our study employed the fixed-dose MTX regimen. This choice was based on its alignment with our institutional practice and supported by previous studies reporting favorable efficacy outcomes [7]. Additionally, we carefully monitored individual patient factors, including body weight, to ensure the appropriateness of the treatment strategy.

The advantage of this experiment is that the study was rigorously designed, since trophoblastic tumours are rare tumours, but the incidence is higher in China than in other regions, and the study will recruit patients from multiple centres to improve the speed of enrolment and represent more territories. This experiment will provide high-quality clinical evidence for the preferred chemotherapy regimen for patients with low-risk trophoblastic tumours.

Several previous studies have shown that among low-risk patients with a score of 0–6, the rate of single-drug resistance increases in patients with a prognostic score of 5–6, so only patients with a score of 0–4 will be included in this study. In addition, the study will make corresponding observations on the safety of the drug and the effect of the drug on ovarian function.

In line with current guidelines, our study protocol includes 2–3 cycles of consolidation chemotherapy for low-risk GTN patients, with the specific number of cycles determined based on individual patient factors. It is worth noting that the study by Lybol et al. [10] highlighted the association between consolidation chemotherapy and relapse rates. While our primary focus is to compare the efficacy of the two treatment regimens, we also recognize the importance of assessing relapse outcomes. In our subsequent analysis of relapse, we will incorporate the number of consolidation cycles as a variable of interest. This will allow us to comprehensively evaluate the impact of consolidation therapy on treatment outcomes. By addressing the relationship between consolidation chemotherapy and relapse rates in our study, we aim to contribute to the understanding of optimal treatment strategies for low-risk GTN patients.

There are certain limitations that should be acknowledged. Firstly, our study utilized the EORTC QLQ-C30 questionnaire, which, although widely accepted and extensively used in studies involving gynecologic cancer patients, may not capture all specific aspects of gestational trophoblastic neoplasia (GTN) patients. GTN presents unique challenges and experiences that may require disease-specific quality of life assessment tools. Unfortunately, there is currently a lack of specifically validated Chinese questionnaires designed for GTN. Secondly, the assessment of quality of life is subjective and influenced by various factors, including individual perceptions and cultural differences. Despite efforts to minimize bias through rigorous methodology and standardized administration, subjective responses may still be subject to inherent limitations. Lastly, our study was conducted in a specific population and setting, which may limit the generalizability of our findings to other patient populations or healthcare systems. It is essential for future research to address these limitations and develop comprehensive, disease-specific tools to assess the quality of life in GTN patients.

## Data Availability

The datasets generated and analysed during the current study are not publicly available due to confidentiality concerns but are available from the corresponding author on reasonable request. The protocol of the trail will be published when the study is halfway through and is confirmed that no modification is required.

## Abbreviations

Act-D: Actinomycin D
AMH: Anti-Mullerian hormone
CR: Complete remission
CRF: Case report form
GTN: Gestational trophoblastic neoplasia
hCG: Human chorionic gonadotropin
MTX: Methotrexate
NCCN: National Comprehensive Cancer Network
RCTs: Randomized clinical trials
